# On the juice? *Trypanosoma cruzi* vectorial-oral outbreak investigation in a semi-arid rural area of Brazil

**DOI:** 10.1186/s13071-025-07198-9

**Published:** 2025-12-29

**Authors:** Gilmar Ribeiro, Juliana Ribeiro Trabuco Frota, Nathália Paixão de Sousa Silva, Bárbara Meneses Oliveira Barbosa, Luana Sampaio Rios, Luís Cláudio Gualberto da Silva, Renato Freitas de Araújo, Felicidade Mota Pereira, Cristiane Medeiros Moraes de Carvalho, Niamh Ellen Tiernan, Claudilson Bastos, Tycha Bianca Sabaini Pavan, Fred Luciano Neves Santos, Marcia C. Castro, Wildo Navengantes, Rodrigo Gurgel-Gonçalves, Eric Dumonteil, Claudia Herrera, Mitermayer G. Reis

**Affiliations:** 1 Pathology and Molecular Biology Laboratory, Gonçalo Moniz Institute (IGM) - FIOCRUZ-BA, Salvador, Bahia Brazil; 2 Chagas Disease Fiocruz Translational Program (Fio-Chagas), Oswaldo Cruz Foundation - Rio de Janeiro (FIOCRUZ-RJ), Rio de Janeiro, Brazil; 3 North Center Health Regional Center, SESAB, Jacobina, Bahia Brazil; 4 Epidemiological Surveillance Coordination, Serrolândia, Bahia Brazil; 5 Strategic Health Surveillance Information Center (CIEVS), North Center Health Regional Center, Jacobina, Bahia Brazil; 6 South Regional Health Center, Ilhéus, Bahia Brazil; 7 Central Public Health Laboratory of the State of Bahia (LACEN), Salvador, Bahia Brazil; 8 Epidemiological Surveillance Directorate (DIVEP), Salvador , Bahia Brazil; 9 Bahia State University, Salvador, Brazil; 10 Advanced Laboratory of Public Health, Gonçalo Moniz Institute (IGM), FIOCRUZ-BA, Salvador, Bahia Brazil; 11 Department of Global Health and Population, Harvard T.H. Chan School of Public Health, Boston, MA USA; 12 National Institute for Health Technology Assessment, Porto Alegre, Brazil; 13 Postgraduate Program in Public Health, University of Brasília, Brasília, Brazil; 14 Laboratory of Medical Parasitology and Vector Biology, Faculty of Medicine, University of Brasília, Brasília, Brazil; 15 Celia Scott Weatherhead School of Public Health and Tropical Medicine, Department of Tropical Medicine and Infectious Disease, Tulane University, Louisiana, USA; 16 Faculty of Medicine of the Federal University of Bahia (FMB-UFBA), Avenida Reitor Miguel Calmon, S/N, Vale Do Canela, Salvador, Bahia Brazil; 17 Yale University, Connecticut, USA

**Keywords:** Acute Chagas disease, Chagas disease, *T. cruzi*, Oral outbreak, Brazil

## Abstract

**Background:**

Oral transmission of Chagas disease has been registered in rural and periurban areas of South America. In Brazil, outbreaks have involved food, sugar cane juice, water, or soup contaminated with infected triatomines or their feces. Here, we report an investigation into an acute Chagas disease outbreak in a rural area of the municipality of Serrolândia, in the semi-arid region of Bahia, Brazil.

**Methods:**

We conducted a descriptive study based on primary care information, epidemiological evaluation, entomological surveillance, and molecular analysis. The investigation began after the death of a 12-year-old child. The study population included triatomines, animals (one opossum, three cats, and two dogs), and a human family of five individuals. We performed serological diagnosis of family members, *Trypanosoma cruzi* molecular detection and genotyping in collected samples, and triatomine blood meal analysis.

**Results:**

Among the five family members, four tested positive for acute Chagas disease. All affected individuals reported ingesting acerola juice from fruits grown on their property, except for case 5, who did not consume the juice or fresh fruit and tested negative for *T. cruzi*. During the investigation, we captured 21 triatomines and collected blood samples from sylvatic and domestic animals. TcI haplotypes show a close relationship between the parasites found in vectors and those detected in a single human case and in the wild reservoir captured, respectively, reinforcing the vector/oral transmission hypothesis and the maintenance of the *T. cruzi* anthropozoonotic cycle in the region.

**Conclusions:**

This study describes the investigation of an acute Chagas disease outbreak in the Serrolândia municipality, and based on that, we conclude that the infection occurred through the vector/oral route via ingestion of *T. cruzi*-contaminated fresh acerola fruit or juice. Our findings underscore the need for improved surveillance and preventive measures in areas vulnerable to Chagas disease.

**Graphical abstract:**

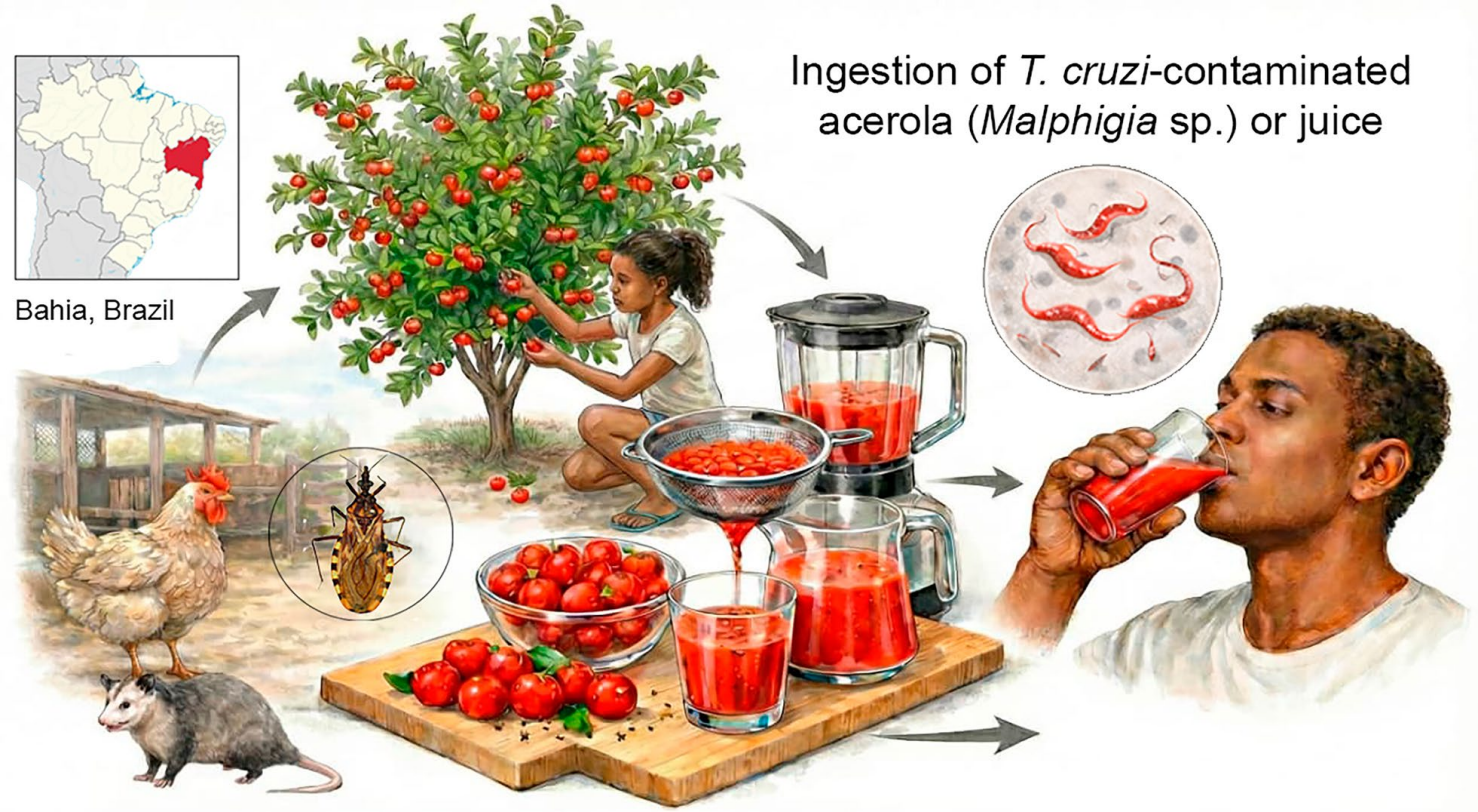

**Supplementary Information:**

The online version contains supplementary material available at 10.1186/s13071-025-07198-9.

## Background

Chagas disease (CD) is a potentially fatal infection caused by *Trypanosoma cruzi* (Chagas, 1909), a protozoan parasite (Kinetoplastida: Trypanosomatidae). In Latin America, 5–7 million CD-infected individuals are estimated [1], and in Brazil, the estimated range is 1–2 million, corresponding to approximately 1–2.4% of the population [2]. CD is classified as a neglected tropical disease (NTD), and the parasite can be transmitted through several routes, including triatomine bugs (Hemiptera: Reduviidae), blood transfusion, organ transplantation, vertical (congenital) transmission, and ingestion of contaminated food [3].

*Trypanosoma cruzi* oral transmission occurs when a person ingests food contaminated with triatomine (vectorial-oral) or mammalian (classic-oral) parasites, and both routes play an important role in transmission [4]. Although vectorial/oral transmission is less common than vectorial transmission, this route can lead to outbreaks. In recent years, multiple cases of oral transmission have been reported in Latin America [5–8], including Brazil [9–12]. These events emphasize the urgent need for improved hygiene practices, vector control, and effective health education campaigns to prevent further events.

The largest *T. cruzi* oral outbreak occurred in a school in Caracas, Venezuela, where 103 people were infected after exposure to guava juice (*Psidium guajava*) contaminated with *T. cruzi*, likely transmitted by the triatomine species *Panstrongylus geniculatus* [5, 7]. In Bahia, Brazil, an outbreak in the city of Macaúbas resulted in 13 infections following the consumption of water during a party, involving *Triatoma sordida* [12]. Between 1965 and 2022, 33 outbreaks of orally transmitted *T. cruzi* were recorded, comprising over 1000 cases, with Brazil reporting the second-highest number of cases [13]. Here, we report an investigation into an acute Chagas disease outbreak in a rural area of the municipality of Serrolândia, in the semi-arid region of Bahia, Brazil.

## Methods

### Study area and design

**Fig. 1:**
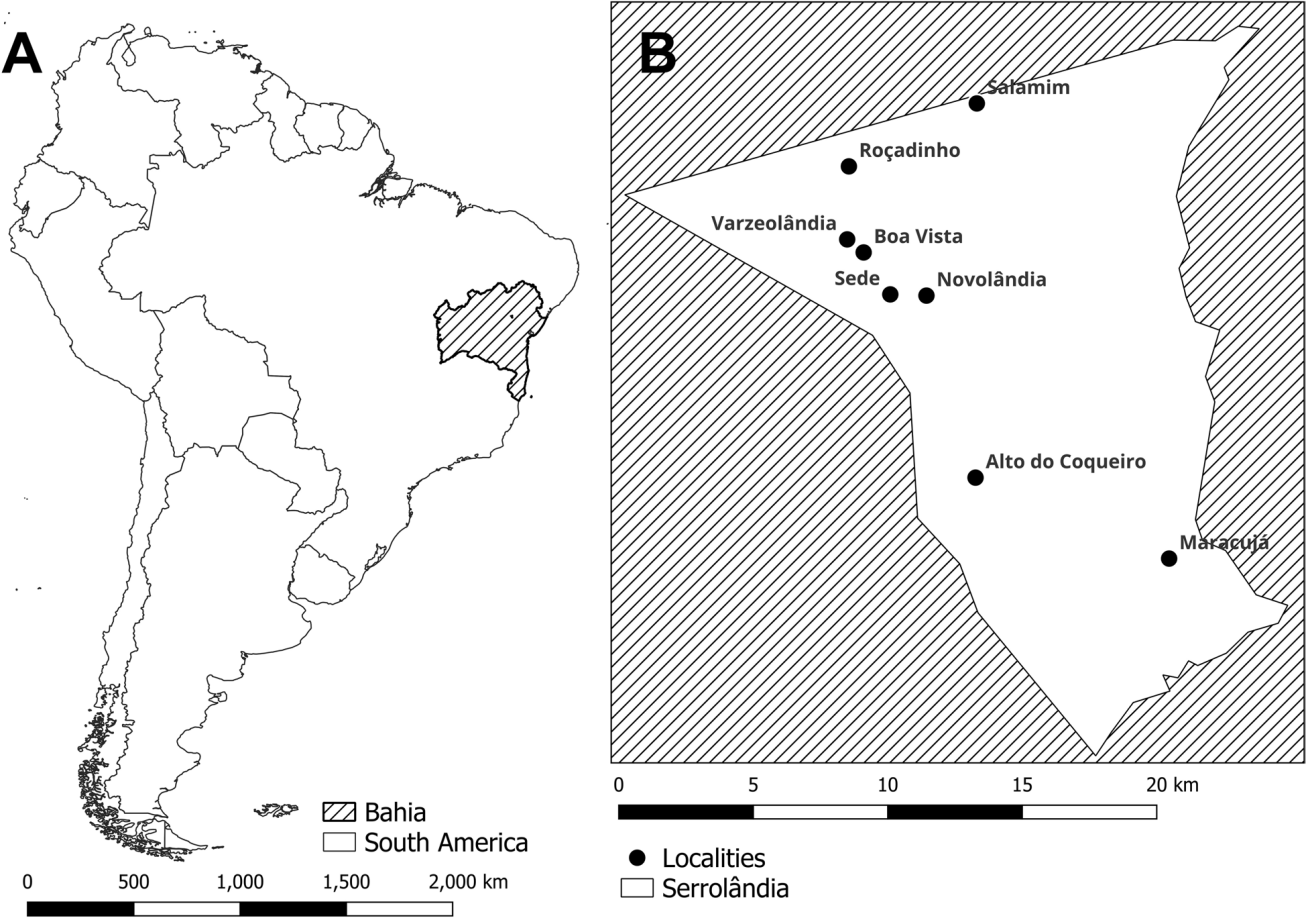
Study area. **A** South America, Brazil and Bahia. **B** Serrolândia municipality and rural areas locations

We conducted a descriptive study to investigate a family outbreak of acute oral Chagas disease in the municipality of Serrolândia (11°24′57″S, 40°18′07″O), located in a semi-arid region characterized by a scrub forest biome, in the Central-North region of Bahia, within the Piedmont Diamantina Identity Territory. Serrolândia has approximately 13,335 inhabitants and is among the lowest-ranked municipalities in Bahia (355th out of 417) and in Brazil (5302nd out of 5570). The municipality comprises an urban center and seven rural settlements: Alto do Coqueiro, Boa Vista, Maracujá, Novolândia, Roçadinho, Salamim, and Varzeolândia (Fig. 1).

### Study population

The study population included triatomines, animals (one opossum, three cats, and two dogs), and a human family of five individuals. All family members who were exposed participated in this investigation.

### Serological diagnosis

Approximately 5 ml of peripheral blood were collected from each participant. Samples were allowed to clot at room temperature for 30 min, then centrifuged at 3000 × *g* for 15 min. The resulting serum was separated and stored at −20 °C until analysis. Serum samples were sent to the Central Public Health Laboratories of Minas Gerais (LACEN-MG) and Bahia (LACEN-BA) for serological testing to detect anti-*T. cruzi* IgM and IgG antibodies, respectively. IgM antibodies were detected using an in-house indirect immunofluorescence assay following standardized procedures. Glass slides coated with 10 µL of *T. cruzi* Y-strain epimastigotes (1.3 × 10^3^ parasites/well) served as the antigen source. Serum samples were screened at 1:10 and 1:20 dilutions in PBS (pH 7.2), and 10 µl of each dilution was applied to individual wells. Slides were incubated in a moist chamber at 37 °C for 30 min, washed three times in PBS for 5 min each, and then air-dried. Antigen–antibody complexes were visualized by applying 15 µL of fluorescein isothiocyanate (FITC)-conjugated anti-human IgM (Anti-Human IgM, Bethyl, USA) diluted in PBS containing 0.01% Evans blue. After a second 30-min incubation at 37 °C, slides were washed again, mounted in buffered glycerol (pH 9.0), and covered with coverslips. Reactions were examined under a fluorescence microscope at 400 × magnification. Green fluorescence in parasites was interpreted as a reactive result, whereas the presence of red, nonfluorescent parasites indicated nonreactive sera. Samples with weak or questionable fluorescence were retested. Reactive sera were titrated to end point dilutions up to 1:320. Each run included positive (1:10 or 1:320) and negative (< 1:10) control sera for assay validation. Titers > 1:40 were considered reactive; titers of 1:10–1:20 were classified as indeterminate; and titers < 1:10 were interpreted as nonreactive. Total IgG anti-*T. cruzi* antibodies were detected using two immunoassays based on different methodological principles: an enzyme immunoassay (anti-Chagas SYM, Vyttra Diagnósticos, São Paulo, Brazil) and an electrochemiluminescence assay (Elecsys Chagas, Roche Diagnostics, Mannheim, Germany). All procedures followed the manufacturer’s instructions. In cases of discordant IgG results, a third confirmatory test was performed using the indirect immunofluorescence assay (IIF Chagas, Bio-Manguinhos, Fiocruz, Rio de Janeiro, Brazil).

### *Trypanosoma cruzi* molecular detection and genotyping

DNA was purified from blood samples of mammalian hosts and human cases, and from the abdomen of triatomines for *T. cruzi* molecular detection. Two end point PCR assays were performed: one targeting nuclear satellite DNA with primers TcZ1 and TcZ2, and another targeting kinetoplast DNA with primers 121–122 [14, 15]. Samples were considered PCR-positive if at least one assay yielded a positive result. Positive samples were further genotyped to assess parasite diversity based on the mini-exon marker, as previously described [16, 17]. Briefly, mini-exon PCR amplicons were end-repaired, indexed, and pooled for sequencing on an Illumina MiSeq platform. FASTQ reads were trimmed to remove index sequences and adapters, then competitively mapped to mini-exon sequences representing all seven *T. cruzi* discrete typing units (DTUs). single-nucleotide polymorphisms (SNPs) were identified using Free-Bayes to generate unique haplotypes, which were aligned with reference sequences. Phylogenetic trees were generated using FastTree to evaluate relationships among sequences from human cases, vectors, and mammalian hosts. A TCS haplotype network was also generated using PopArt [19, 20, 21]. Newly generated *T. cruzi* sequences from this investigation are available in GenBank under accession numbers PV340862-PV340930.

### Triatomine blood meal analysis

The blood meal sources were identified in triatomines by sequencing 12S gene amplicons from the triatomine gut DNA samples [18]. Briefly, these amplicons were pooled with mini-exon amplicons for simultaneous sequencing on the MiSeq platform, and raw reads were mapped to a human 12S reference sequence. Genus and species identification were performed using MegaBlast with a sequence identity threshold of > 97% [18].

### Data analysis and geoprocessing

Data were tabulated using spreadsheets, and geoprocessing analyses were performed using Qgis^®^ software version 3.42.2 (https://qgis.org/download/).

## Results

### Timeline of the outbreak

This study involved five family members: case 1 (the index case), a 12-year-old girl; case 2, the 53-year-old father; case 3, the 42-year-old mother; case 4, the 17-year-old son; and case 5, the 5-year-old daughter. Detailed timeline of main activities during an acute Chagas disease outbreak and the clinical evaluation of each case are shown in Additional file 1. Text S1–S2.

### January 2023: initial symptoms and case 1’s death

- All family members except case 5 (who was asymptomatic) developed nonspecific symptoms (fever, myalgia, rash). They sought medical care at a local Family Health Unit, where dengue was suspected, and treatment began.
- The symptoms of cases 1 and 2 worsened, leading to further consultations and intravenous hydration at Serrolândia Municipal Hospital.
- Case 1’s condition significantly deteriorated, presenting severe dyspnea, facial edema, and jaundice. On 27 January, she returned to the Municipal Hospital with low oxygen saturation; a chest x-ray revealed pleural effusion and cardiomegaly.
- On 28 January, case 1 was transferred to Santo Antônio Hospital (Salvador, Bahia) with respiratory distress, filiform pulses, abdominal pain, and mild lower limb edema (+ /IV). Volume expansion and vasoactive medications were administered. Imaging showed pneumonia with right pleural effusion, ascites, and hepatic abnormalities. Thoracentesis drained 1030 mL of right hemothorax and 300 mL of citrine ascitic fluid.
- Case 1 was intubated, developed bloody tracheal secretions, suffered cardiac arrest unresponsive to 30 min of CPR, and died on 29 January, 2023.

### February–April 2023: CD confirmation and family testing

- January 30: Serrolândia Epidemiological Surveillance (VIEP) reported case 1’s death to the Strategic Information Center for Health Surveillance (CIEVS), informing the Chagas Disease Program and NRS CN technical references.
- Suspecting Chagas disease (Serrolândia is a medium-risk area for vector-borne *T. cruzi* transmission), VIEP requested testing of case 1’s stored blood (LACEN-BA). Initial tests (9 March) were positive for ELISA, electrochemiluminescence, and indirect immunofluorescence (IIF) IgG. Confirmatory re-evaluation (28 March) yielded positive IgM, ELISA, IIF IgG, and indirect hemagglutination assay (IHA) results.
- VIEP coordinated with LACEN-BA to test other family members:
- Case 3 (sampled 19 January): Positive for ELISA, IIF (IgG and IgM), and HAI (7 February).
- Case 2 (sampled 28 March): Positive for ELISA (IgG and IgM) and IHA (11 April).
- Case 4 (sampled 28 March): Positive for ELISA, IIF (IgG and IgM), and HAI (11 April). Case 4 subsequently showed worsening health with cardiac involvement, pericardial effusion, dyspnea, severe lower limb edema (+ +  + /IV), and mild jaundice (+ /IV).
- Case 5 remained asymptomatic and seronegative.
- Despite the serologically confirmed Chagas disease cases, the family’s grief following case 1’s death delayed the start of the epidemiological investigation.

### 18–21 April, 2023: environmental investigation

- The investigation of the family’s urban and rural houses began on 18 April.
- Urban house: no evidence of triatomine presence or *T. cruzi* transmission was found. The home was clean with intact plaster walls and ceiling, and a healthy pet dog was present (Additional file 2: Supplementary Fig. S1).
- Parent interviews revealed that case 1 frequently consumed fresh acerola (*Malpighia* sp.) fruits and juice from their rural property, often unwashed, picked from trees and the ground. Case 5 disliked acerola fruits and did not consume any. At this time, case 3 was asymptomatic; case 2 had mild lower limb edema and jaundice.
- Rural house (Fazenda Barrocas): the investigation shifted to the family’s rural house (Additional file 2: Supplementary Fig. S2). Five housing units were surveyed over 3 days. *Triatoma pseudomaculata* infestation was identified in one peridomestic area, with a colony found in a chicken coop 25 m from the house.
- A thorough house inspection (including chemical agents to dislodge insects) revealed triatomine presence (feces, exuviae, a bloodstain on the couple’s mattress suggestive of a crushed triatomine, and a dead adult *T. pseudomaculata* specimen in the bed frame). The family reported spending several days there in December 2022, consuming acerola juice and fresh fruit from backyard trees. The small rural house had cracked, gapped plaster walls, potential triatomine shelters (Additional file 2: Supplementary Fig. S2).
- The chicken coop (25 m away) yielded seven adult *T. pseudomaculata* specimens (Additional file 2: Supplementary Fig. S3) and 14 nymphs of various developmental stages (Table 1). Blood samples from domestic animals (three cats, two dogs) and one opossum were collected from the brachial vein; no animals were sacrificed. All live triatomine specimens and animal blood samples were sent to Fiocruz-BA and Tulane University for molecular analysis and *T. cruzi* genotyping.

**Table 1:** Samples and laboratory tests of Serrolândia *T. cruzi* oral outbreak, Bahia, Brazil, 2023

Field note	ID	Sample	DNA concentration Nanodrop	DNA Quality Nanodrop 260/280	DNA concentration Qubit	Paras^1^	Serol^2^	PCR^3^	TCI hap
T01	T1	*T. pseudomaculata*—Adult M	289	1.52/5.7	500	−	NA	+	1,2,3,4
T01	T2	*T. pseudomaculata*—Adult M	404	1.41/8.0	453	+	NA	+	1,2,3,4,5,6,7,8
T01	T3	*T. pseudomaculata*—Adult F	196	1.93/3.9	374	−	NA	+	1,2,3,4
T01	T4	*T. pseudomaculata*—Nymph 5	190	1.90/3.8	168	−	NA	−	NA
T01	T5	*T. pseudomaculata*—Nymph 4	199	1.89/3.9	329	−	NA	+	1,2,3,4
T05	T6	*T. pseudomaculata*—Adult M	153	1.95/3.0	471	−	NA	+	1,2,3,4
T05	T7	*T. pseudomaculata*—Nymph 4	258	9.95/5.5	368	−	NA	+	NA
T05	T8	*T. pseudomaculata*—Nymph 3	141	2.07/2.8	229	−	NA	+	1,2,3,4
T05	T9	*T. pseudomaculata*—Nymph 3	135	2.19/2.7	218	ND	NA	+	1,2,3,4
T04	T10	*T. pseudomaculata*—Adult M	152	1.97/3.0	261	−	NA	+	1,2,3,4
T04	T11	*T. pseudomaculata*—Adult F	116	2.00/2.33	213	+	NA	+	1,2,3,4
T04	T12	*T. pseudomaculata*—Adult M	157	1.71/3.15	345	+	NA	+	1,2,3,4,
T04	T13	*T. pseudomaculata*—Nymph 5	201	1.63/4.03	52.6	+	NA	+	1,2,3,4
T04	T14	*T. pseudomaculata*—Nymph 5	219	1.72/4.39	131	−	NA	+	1,2,3,4
T04	T15	*T. pseudomaculata*—Nymph 3	261	2.02/5.22	416	−	NA	+	1,2,3,4
T04	T16	*T. pseudomaculata*—Nymph 3	138	2.02/2.77	154	−	NA	+	1,2,3,4
T04	T17	*T. pseudomaculata*—Nymph 2	156	1.94/3.12	320	ND	NA	+	1,2,3,4
TSN1	T18	*T. pseudomaculata*—Nymph 3	210	1.77/4.21	369	−	NA	−	NA
TSN2	T19	*T. pseudomaculata*—Nymph 4	490	1.77/5.82	380	−	NA	−	NA
NA	T20	Internal DNA extraction control	0.27	1.70/0.35	0.50	NA	NA	−	NA
A1	B1	Cat (Yellow)	15.5	1.70/0.30	49.2	NA	ND	−	NA
A2	B2	Cat (Black)	32.3	1.77/0.64	129	NA	ND	−	NA
A3	B3	Cat (Tabby)	46.5	1.80/0.92	272	NA	ND	−	NA
A4	B4	Opossum (*Didelphis aurita*)	34.8	1.94/0.69	102	NA	ND	+	1,2,3,4
A5	B5	Dog	69.3	1.94/1.31	386	NA	ND	−	NA
A6	B6	Dog	55.6	1.72/1.11	76.7	NA	ND	−	NA
C1	B7	Case 1	32.7	2.00/0.65	230	NA	+	−	NA
C2	B8	Case 2	55.5	1.91/1.11	289	NA	+	+	1
C3	B9	Case 3	79.3	1.85/1.58	570	NA	+	−	NA
NA	B10	Internal DNA extraction control	0.1	1.80/0.00	0.84	NA	+	−	NA
BST	B11	Bloodstain cloth	17.5	1.70/0.30	52.2	NA	NA	−	NA

*ID* = Sample ID number; ^**1**^ = Parasitological examination of fresh triatomine feces samples; ^**2**^ = IgM/IgG serological tests; ^**3**^ = Conventional PCR results; *TcI hap* = *T. cruzi* TcI Discrete Typing Unit genetic classification haplotypes. ND, Not done; NA, not applicable

### Molecular evaluation of *T. cruzi*

**Fig. 2:**
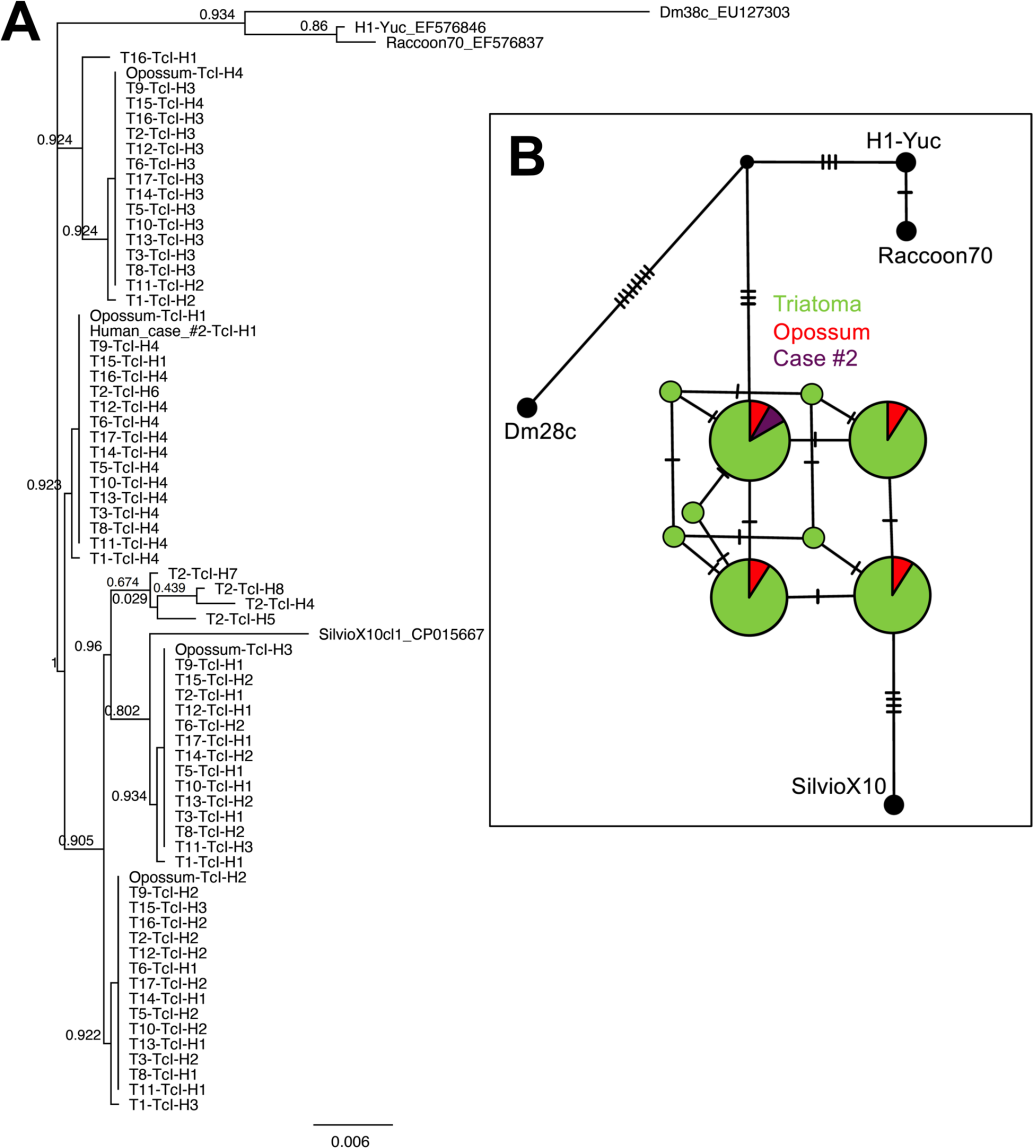
Phylogenetic analysis of *T. cruzi* sequence diversity and haplotype network. **A**. Phylogenetic tree of *T. cruzi* mini-exon sequences based on approximately maximum likelihood in FastTree, using a generalized time-reversible (GTR) model of nucleotide evolution. Numbers on nodes indicate bootstrap support from 1000 replicates based on the Shimodaira–Hasegawa test. Genbank accession numbers are indicated for reference sequences. **B** Templeton, Crandall, and Sing (TCS) haplotype network elaborated in PopArt based on *T. cruzi* mini-exon sequences. Circles represent sequence haplotypes, with their size proportional to the number of sequences, and ticks on the branches indicate the number of mutations between two haplotypes. Haplotypes are color coded for triatomine, opossum, or human case origin. Sequences from *T. cruzi* reference strains are indicated in black

**Fig. 3:**
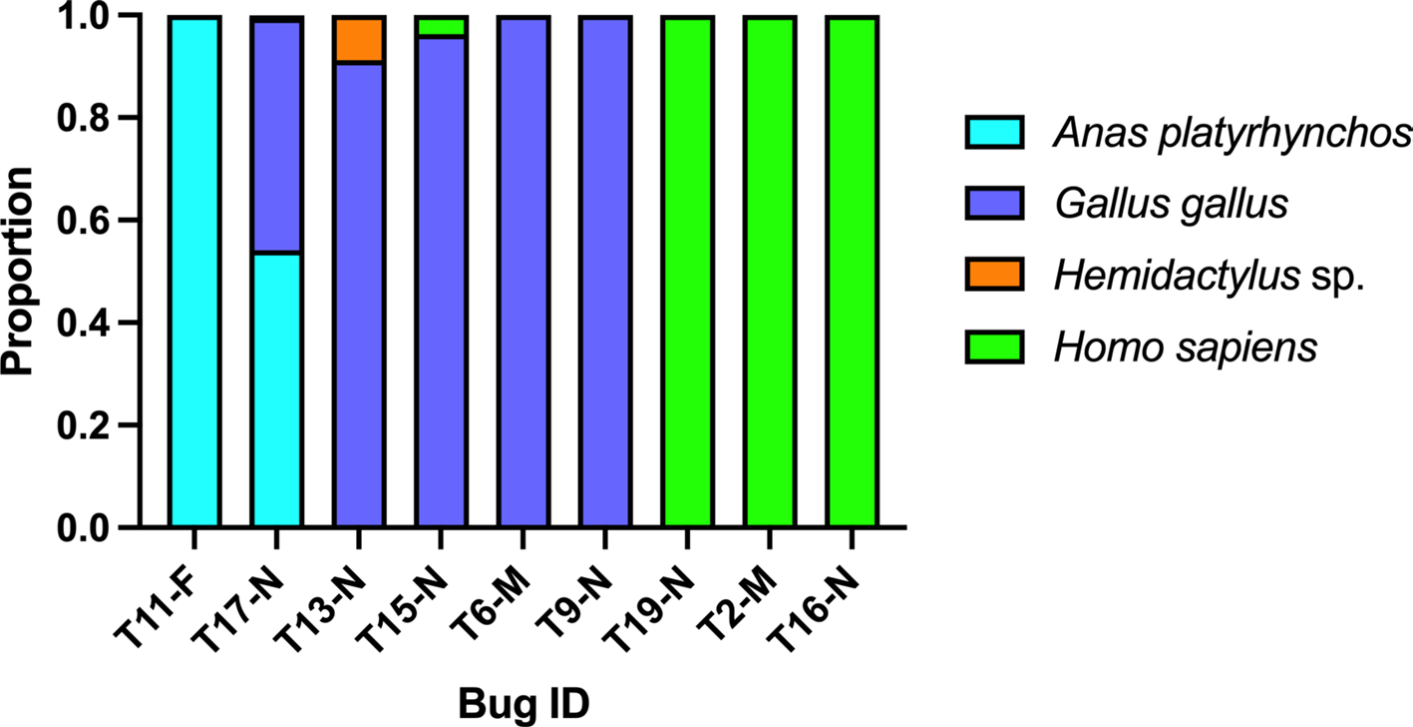
*Triatoma pseudomaculata* blood meal sources. The proportion of blood meal sources is shown for individual bugs. **F** female, **M** male, **N** nymph

PCR analysis showed that 15/19 (79%) of triatomines were infected, with 7/7 (100%) adults and 8/12 (67%) nymphs infected (Table 1). Two dogs and three cats were negative, but one opossum tested positive for *T. cruzi* DNA, as did case #2 from the outbreak (Table 1). Phylogenetic analysis of *T. cruzi* sequences from the positive samples indicated the presence of various TcI haplotypes, most of which were shared among triatomines, the opossum, and human case #2, suggesting a common origin of the parasite circulating in this habitat for food contamination. These TcI parasites were more closely related to the SilvioX10 reference strain from Brazil rather than strains from Colombia (Dm38c) or Mexico (H1-Yuc) (Fig. 2). Further analysis of *T. pseudomaculata* blood meal sources showed a predominant feeding on chickens and humans, and to a lesser extent on ducks (*Anas platyrhynchos* Linnaeus, 1758) and lizards (*Hemidactylus* spp.) (Fig. 3).

## Discussion

This study describes an investigation into an outbreak of orally transmitted *Trypanosoma cruzi* infection in Serrolândia, Bahia, Brazil, involving five individuals from the same family. Four individuals were infected and exhibited symptoms of acute Chagas disease, with one fatality. Epidemiological, entomological, molecular biology analysis, and clinical evidence suggest that transmission occurred via the vectorial-oral route, likely through the ingestion of fresh fruits or acerola (*Malpighia sp.*) juice contaminated with *T. cruzi*. Notably, in case 5, the only uninfected family member did not consume acerola fruit.

Orally transmitted Chagas disease highlights a concerning link to food insecurity. The ingestion of *T. cruzi*-contaminated food in areas where triatomine bugs and natural reservoirs such as *Didelphis* are present makes up a major risk factor for transmission. Both insects and opossums may excrete over fresh food or be inadvertently crushed during food preparation, contaminating food and beverages with *T. cruzi* parasites [13]. In addition, the consumption of bushmeat or wild animals from unregulated and uninspected sources may further increase exposure to *T. cruzi*. Raising awareness about the importance of food hygiene and implementing strict control measures in food production and distribution, particularly for commercialized products, are essential strategies to reduce the incidence of orally transmitted Chagas disease [22, 23].

In primary healthcare settings, a key challenge is accurately identifying *T. cruzi*-infected patients, particularly those in the acute phase of the disease [24]. The initial symptoms often resemble other infectious diseases, leading to diagnostic delays and missed opportunities for timely medical intervention. The acute phase may present nonspecific symptoms such as fever, malaise, headache, and myalgia, which can be easily misattributed to other conditions. Furthermore, limited awareness among healthcare professionals regarding the clinical presentation of acute Chagas disease contributes to misdiagnosis, prolonged patient suffering, and potential further transmission [25].

As reported by Maroto et al. [26], coinfections, which can lead to fatal multiorgan and systemic failure, represent a significant challenge in clinical practice, particularly when involving Chagas disease and other infectious agents. In such cases, the use of steroids may worsen the infection caused by *T. cruzi*. This challenge is often compounded by difficulties in isolating the pathogens, overlapping symptoms, and the absence of specific diagnostic protocols in regions affected by emerging and reemerging diseases. As a result, the limited understanding of these coinfections and their clinical implications hinders timely and effective patient management.

In Brazil, Chagas disease is a legally notifiable condition requiring mandatory reporting of all suspected acute cases. However, mandatory notification of chronic Chagas disease cases was implemented only in 2023. Despite this, a low index of clinical suspicion, exacerbated by inadequate access to information, has led to under-reporting of cases and a misleading perception that the disease burden is lower than it is. To address this, continuous education of healthcare professionals and public health awareness campaigns are crucial. Such initiatives can enhance community engagement and improve case detection and management across all stages of Chagas disease patients [25, 27].

The triatomine bug registered in this outbreak was *T. pseudomaculata*, a native species widely distributed in Brazil, particularly in the semi-arid Caatinga biome of the Northeast region [29]. The presence of *T. pseudomaculata* inside households has been documented in both Bahia and Minas Gerais. In the Jequitinhonha Valley, this species has been found in domiciliary environments associated with bats [28]. In Bahia, chemical control measures have had limited impact on reducing *T. pseudomaculata* populations, and this species was among the most frequently captured triatomines in the state between 2006 and 2019 [30]. *T. pseudomaculata* has the potential to colonize human dwellings, and a natural *T. cruzi* infection rate of approximately 12% [30]. Its occurrence is high in households near preserved forests with natural ecotypes, including bird nests, trees, and palm groves [32]. The association of *T. pseudomaculata* with an outbreak of acute Chagas disease underscores its epidemiological significance in the semi-arid area.

From a One Health perspective, deforestation and habitat fragmentation directly affect the colonization dynamics of native triatomine [33, 34] and the prevalence of sylvatic *T. cruzi* reservoirs, such as the opossum *Didelphis aurita* [35]. These environmental disturbances facilitate the spread of Chagas disease by increasing vector–human contact. In addition, the unregulated exploitation of native vegetation reduces the availability of natural reservoirs and shelters for triatomine bugs, forcing them to seek alternative refuges in human dwellings, where food and shelter are readily available [36, 31]. Artificial lighting has also been shown to attract triatomines to houses, particularly during twilight hours and specific seasonal periods [37]. The primary limitation of this investigation was the inability to analyze the juice and fruits consumed by the affected family, which were likely the major sources of infection.

To ensure adequate clinical management of infected patients, implementing the Chagas disease control program at the municipal level is essential. This includes ongoing training for healthcare professionals, targeted health communication campaigns, and active involvement of municipal authorities. Such measures are critical for enhancing community awareness and facilitating the early detection of acute cases.

The Brazilian public health system (SUS) could detect and treat patients with acute *T. cruzi* infection and provide healthcare support to affected individuals. However, given the nonspecific clinical signs and symptoms of acute Chagas disease, integrating rapid diagnostic tests into SUS would be an accessible and efficient confirmatory tool at the municipal and regional levels, helping to prevent the misinterpretation of clinical manifestations, reduce diagnostic delays, and enable timely and accurate treatment of *T. cruzi*-infected patients. Furthermore, we highlight that severe cases of acute Chagas disease may be associated with coinfections, as demonstrated in this study, which reported the simultaneous presence of Chagas disease and Chikungunya. This scenario underscores the importance of integrated surveillance for more effective management in contexts with multiple infections, as the administration of steroids may worsen *T. cruzi* infection.

## Conclusions

Given the temporal association, epidemiological link, entomological research findings, the clinical presentation of the cases, and similarity of *T. cruzi Tc*I haplotypes among samples, we indicate that the acute Chagas disease outbreak occurred through the vectorial-oral route, likely via the ingestion of fresh acerola (*Malpighia*sp.) fruit or fruit juice contaminated with *T. cruzi*. Our findings underscore the need for improved surveillance and preventive measures in areas vulnerable to Chagas disease.

## Supplementary Information

Supplementary file 1.

Supplementary file 2.

## Data Availability

Data supporting the main conclusions of this study are included in the manuscript.
