# Supplementary material for: On the juice? Trypanosoma cruzi vectorial-oral outbreak investigation in a semi-arid rural area of Brazil
Source: Parasit Vectors. 2025 Dec 29;19:59. doi: 10.1186/s13071-025-07198-9 (PMC12859902; doi:10.1186/s13071-025-07198-9)
Supplement: Supplementary file 1 [file 13071_2025_7198_MOESM1_ESM.docx]

**Additional file 1: Text S1: T**imeline of main activities during an acute Chagas disease outbreak in Serrolândia, Brazil, 2023.

| **ID** | **Data** | **Activity** |
| --- | --- | --- |
| 1 | 12/01/2023 | Epidemiological surveillance team is informed of possible cases of dengue fever in a family, contact made by the Municipal Hospital of Serrolândia |
| 2 | 13/01/2023 | The endemic control team carried out an active search together with the area's community health agents, performing coastal and focal blocking of the peridomiciliary region |
| 3 | 13/01/2023 | Basic Health Unit is notified of suspected cases and begins monitoring |
| 4 | 16/01/2023 | The endemic control team carried out an active search together with the area's community health agents, performing coastal and focal blocking of the peridomiciliary region |
| 5 | 27/01/2023 | Case 1 was admitted to the Municipal Hospital of Serrolândia with low oxygen saturation, dyspnea, pallor, and asthenia. |
| 6 | 28/01/2023 | Case 1 referred to Hospital Santo Antônio, Salvador |
| 7 | 29/01/2023 | Case 1's death confirmed |
| 8 | 30/01/2023 | Epidemiological surveillance team visit to family members |
| 9 | 31/01/2023 | Carrying out the first imaging tests (chest x-ray) |
| 10 | 03/02/2023 | Requested from Central Laboratory for vials to perform blood cultures on all family members, and a request for serology for Chagas IgM and IgG for Case 1 |
| 11 | 06/02/2023 | Blood culture collection was performed on the father (Case 2), mother (Case 3), and two children, aged 17 (Case 4) and 10 (Case 5) years |
| 12 | 07/02/2023 | First IgG reagent result for Chagas disease (mother - Case 3) and contact made with regional health center-Centro Norte for more information regarding the death investigation |
| 13 | 08/02/2023 | Family consultation with a cardiologist |
| 14 | 10/02/2023 | Family consultation with a doctor at the Basic Health Unit |
| 15 | 14/02/2023 | Negative result obtained for microbial growth by blood culture |
| 16 | 17/02/2023 | Family consultation with a doctor at the Basic Health Unit |
| 17 | 09/03/2023 | Second IgG reagent result for Chagas disease (Case 1) |
| 18 | 21/03/2023 | Deliver the requests for serology tests for Chagas disease to other family members and for evaluation by a doctor at the Basic Health Unit |
| 19 | 27/03/2023 | Visit of the regional health center -Centro Norte with the presence of Primary Care, Epidemiological surveillance team, and Chagas-Endemic reference, to the farm to talk with family members |
| 20 | 31/03/2023 | First IgM reagent result for Chagas disease (Case 1) |
| 21 | 18/04/2023 | First active search on the rural and urban houses for entomological investigation |
| 22 | 19/04/2023 | Second active search on the farm and neighboring houses, laboratory analysis of triatomines found, and conversation with the municipal manager to expand actions to control Chagas disease |
| 23 | 20/04/2023 | Third active search on the farm to collect blood and a natural reservoir of *T. cruzi*, *Didelphis aurita* captured, in addition to spraying throughout the farm territory |
| 24 | 25/04/2023 | Spraying in a residence in an urban area |
| 25 | 25/04/2023 | Initiation of treatment with benznidazole 300 mg/day (Case 2) |
| 26 | 26/04/2023 | Initiation of treatment with benznidazole 300 mg/day (Cases 3 and 4) |
| 27 | 03/05/2023 | Second IgM reagent result for Chagas disease (Case 2) |
| 28 | 04/05/2023 | Third IgM reagent result for Chagas disease (Cases 3 and 4) |
| 29 | 04/08/2023 | Consultation with an infectious disease specialist at Couto Maia, Salvador (Cases 2, 3, and 4) |
| 30 | 09/08/2023 | Municipal epidemiological surveillance team visits patients and requests new laboratory tests |

**Additional file 1: Text S2: clinical evaluation of the Cases**

Case 1 - Index:

A 12-year-old female patient was admitted to Serrolândia Municipal Hospital on January 29, 2023, presenting with dyspnea, vomiting, fever, and cough for approximately two weeks, with worsening clinical conditions. Upon arrival at the emergency room, she exhibited tachypnea, central cyanosis, and dyspnea, with an oxygen saturation of 61% in room air. Physical examination revealed decreased vesicular breath sounds in the right hemithorax, predominantly at the bases. The patient showed clinical improvement after nebulization and hydrocortisone administration. Vital signs: HR: 113 bpm; RR: 30 irpm; SBP: 87 mmHg; DBP: 69 mmHg; Temperature: 36.5°C; O₂ saturation: 93% with nasal cannula. She was treated with ceftriaxone (initiated on January 27, 2023) and hydrocortisone. Laboratory results: Hb: 11.5 g/dL; Hct: 34.7%; Plt: 352,000/mm³; WBC: 14,300/mm³; Seg: 78.5%; Lymph: 15.5%; Urea: 32 mg/dL; Creatinine: 0.69 mg/dL; Glucose: 101 mg/dL; AST: 54 U/L; ALT: 35 U/L; COVID-19 test: non-reactive. Chest X-ray revealed consolidative opacity/atelectasis in the right lower lung field, with veiled costophrenic sinuses. Cardiac area evaluation was impaired. Clinical outcome: death. Positive IgM serology for Chikungunha on February 3, and Positive serology for *T. cruzi* on March 9, 2023.

Case 2:

A 53-year-old male patient reported a history of fever and presented with dyspnea, lower limb edema, and cutaneous jaundice. A cardiological evaluation confirmed pericardial effusion and an enlarged cardiac area on chest X-ray. Treatment for congestive heart failure (CHF) was started. The patient denied any comorbidities. Laboratory results (January 28, 2023): Hb: 11.40 g/dL; Hct: 36.90%; WBC: 5,170/mm³; Seg: 37.1%; Eosin: 0.2%; Lymph: 55.9%; Plt: 223,000/mm³; Fasting glucose: 97 mg/dL; Urea: 37.7 mg/dL; Creatinine: 0.70 mg/dL. Electrocardiogram (March 23, 2023) showed atrial fibrillation, low frontal voltage, and nonspecific changes in ventricular repolarization. Chest X-ray (January 31, 2023) revealed normal lung transparency, free costophrenic sinuses, and a cardiac area within normal limits. Positive serology for *T. cruzi* on April 11, 2023. Following medical evaluation, treatment with oral benznidazole (100 mg every 8 hours for 74 days) was prescribed. During therapy, the patient developed erythematous patches on the trunk, abdomen, and limbs, which resolved after a 10-day course of oral prednisone (5 mg). The patient remains in satisfactory health, under specialist follow-up.

Case 3:

A 42-year-old female patient reported a history of fever and joint pain, followed by lower limb edema and difficulty in mobility. She denied any comorbidities. Laboratory results (January 19, 2023): Hb: 10.4 g/dL; WBC: 7,900/mm³; Plt: 361,000/mm³. Electrocardiogram (February 6, 2023) showed nonspecific changes in ventricular repolarization. Chest X-ray (January 31, 2023) revealed normal lung transparency, free costophrenic sinuses, and a cardiac area within normal limits. Abdominal ultrasound (April 18, 2023) showed no abnormalities. Positive serology for *T. cruzi* on February 7, 2023. After medical evaluation, the patient was treated with oral benzonidazole (100 mg every 8 hours for 67 days). No adverse events were reported during therapy. The patient remains in satisfactory health, under specialist follow-up.

Case 4:

A 17-year-old male patient presented with fever, diarrhea, vomiting, epigastric pain, joint pain, facial and lower limb edema, arrhythmia, and precordial stabbing pain. Laboratory results (April 8, 2023): Hb: 15.3 g/dL; WBC: 4,020/mm³; Plt: 161,000/mm³; TSH: 3.43 mUI/L; AST: 20 U/L; ALT: 19 U/L; Urine albumin: 4.6 g/dL; Fasting glucose: 95 mg/dL. Cyst and parasite examination: negative. Additional tests: Chest X-ray (January 31, 2023) showed normal lung transparency, free costophrenic sinuses, and a cardiac area within normal limits. Urinary system ultrasound (February 4, 2023) and spirometry (February 6, 2023) showed no abnormalities. Abdominal ultrasound (April 18, 2023) was also unremarkable. Positive serology for *T. cruzi* on April 5, 2023. After medical evaluation, the patient was treated with oral benznidazole (100 mg every 8 hours for 60 days). During therapy, he experienced melena episodes, occurring three times daily for five days. The patient remains in stable condition, under cardiology follow-up.

Case 5:

This was the only case that tested negative for *T. cruzi* and did not develop symptoms. Negative serology for *T. cruzi* on April 5, 2023.
