# Supplementary material for: On the juice? Trypanosoma cruzi vectorial-oral outbreak investigation in a semi-arid rural area of Brazil
Source: Parasit Vectors. 2025 Dec 29;19:59. doi: 10.1186/s13071-025-07198-9 (PMC12859902; doi:10.1186/s13071-025-07198-9)
Supplement: Supplementary file 2 [file 13071_2025_7198_MOESM2_ESM.docx]

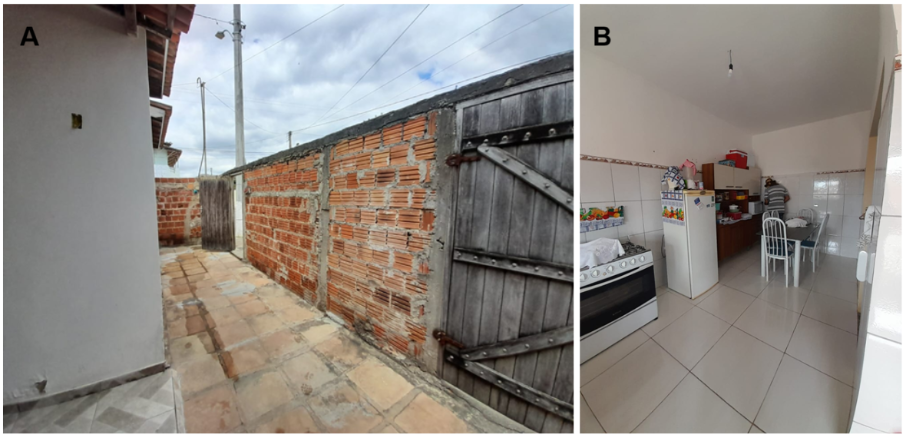


**Additional file 2: Fig. S1**. House located in the urban area of Serrolândia, Bahia, Brazil. A: The front yard and house entrance. B: Kitchen.


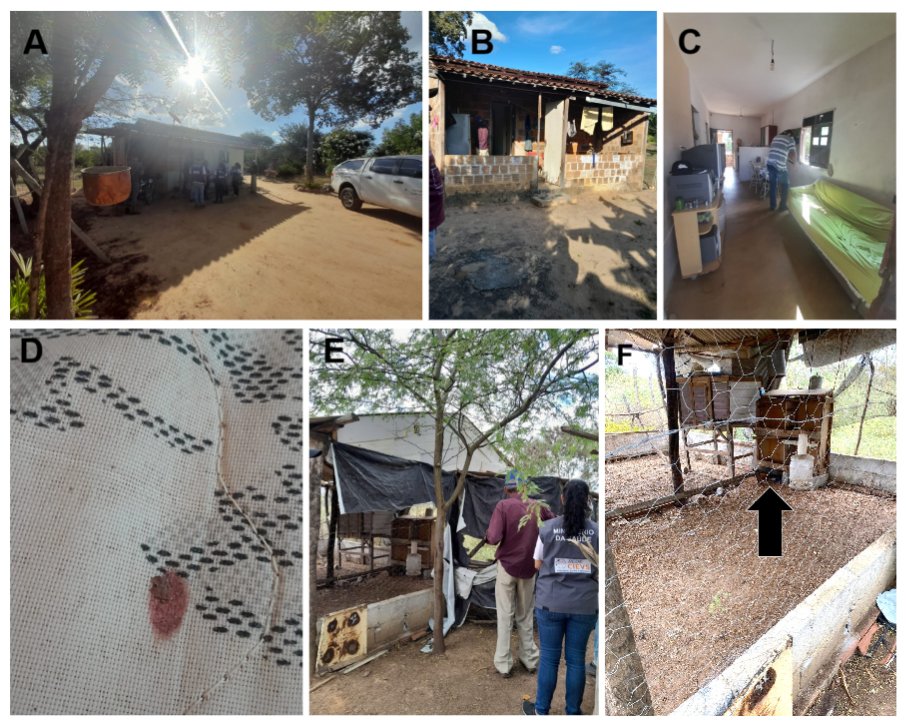


**Additional file 2: Fig. S2.** Investigation in the rural area of Serrolândia, Bahia, Brazil. A: Front of the house. B: Back of the house. C: Lliving room. D: Bloodstain on the couple's mattress. E: Chicken coop located approximately 25 meters from the house. F: The arrow indicates the wooden structure where the chicken nest was located and the triatomines were collected.


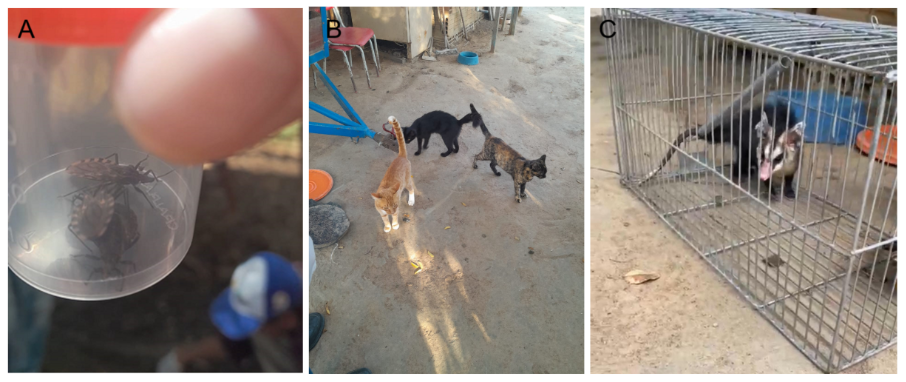


**Additional file 2: Fig. S3** Triatomines and hosts sampled in the rural house in Serrolândia, Bahia, Brazil. A: *Triatoma pseudomaculata*. B: Cats. C: *Didelphis aurita*.
